# Beyond Borders: A Longitudinal Study of Nepali Nurses’ Dreams, Realities, and the Pursuit of a Global Career

**DOI:** 10.1177/15271544251322493

**Published:** 2025-03-13

**Authors:** Animesh Ghimire, Yunjing Qiu

**Affiliations:** 1 School of Nursing and Midwifery, 2541 Monash University, Clayton VIC, Australia; 2 Sustainable Prosperity Initiative Nepal, Kathmandu, Nepal; 3 School of Nursing and Midwifery, 1994 University of Technology Sydney, Ultimo, NSW, Australia

**Keywords:** motivation, developing countries, resilience, workforce

## Abstract

The global migration of nurses, particularly from developing nations like Nepal, is a complex tapestry woven with threads of ambition, sacrifice, and resilience. This longitudinal qualitative study followed the journeys of 17 Nepali nursing graduates, some who embarked on international careers and others who chose to remain in their homeland. Their narratives challenge simplistic notions of “brain drain,” revealing a dynamic interplay of evolving motivations, unexpected opportunities, and the bittersweet realities of pursuing dreams abroad. The study uncovers a stark contrast between the idealized vision of working in “modern, first-world hospitals” and the lived experiences of migrant nurses, highlighting the emotional toll of cultural adjustment and the often-unmet expectations related to financial gains and professional advancement. Yet, amidst these challenges, nurses demonstrated remarkable adaptability, leveraging language acquisition, mentorship, and community building as strategies for integration and resilience. The study also sheds light on the unexpected paths to fulfillment found by those who remained in Nepal, challenging the prevailing narrative that migration is the sole route to success. These findings underscore the urgent need for comprehensive policies and support systems that address both the structural factors driving migration and the individual needs of nurses, fostering a more equitable and sustainable global healthcare workforce.

## Highlights

- Nepali nurses’ migration journeys are complex and defy simplistic narratives of mass exodus.
- The gap between expectations and reality abroad underscores the need for realistic premigration preparation.
- Challenging assumptions, some nurses find rewarding careers within Nepal.
- Emotional factors, not just financial gains, shape migration decisions.
- Migrant nurses demonstrate adaptability and resourcefulness in navigating challenges.

## Introduction

The global migration of healthcare professionals, particularly nurses, is a pressing concern with implications for healthcare systems, economies, and individual well-being. As highlighted in the State of the World's Nursing 2020 report, the global shortage of nurses is a pressing issue (Buchan et al., 2022; World Health Organization [WHO], 2020). This phenomenon is characterized by the movement of skilled nurses from resource-constrained countries to wealthier nations as a means of addressing workforce shortages (McKeown et al., 2023; Stievano et al., 2022). While extensive research has explored the push and pull factors driving migration (Konlan et al., 2023; Laari et al., 2024; Toyin-Thomas et al., 2023), less attention has been given to the evolving motivations, unmet expectations, and adaptation strategies of migrant nurses over time, particularly those from developing countries such as Nepal.

This study builds upon previous research, which explored the factors influencing the migration intentions of undergraduate nursing students in Nepal (Ghimire, Qiu, Thapa, et al., 2024). In that initial investigation, we conducted a qualitative study involving 17 final-year nursing students from two urban colleges. Our findings revealed a unanimous intention to migrate among all participants, driven by a complex interplay of structural and systemic catalysts, personal ambitions, socio-political currents, and economic incentives. Specifically, the study identified four key themes shaping these intentions: “Aspirations Beyond Borders,” highlighting systemic inefficiencies and limitations in the Nepali healthcare system; “Navigating Personal Ambitions,” which underscored the desire for professional growth and the pursuit of opportunities perceived as unavailable within Nepal; “Socio-Political Currents Shaping Career Pathways,” revealing concerns about political instability and its impact on the nursing profession; and “Economic Incentives and Realities,” emphasizing the significant role of financial considerations in migration decision-making.

This longitudinal study follows the same cohort of 17 nurses one year later to examine the evolution of their migration journeys, comparing their initial intentions with their actual experiences, whether they chose to migrate or remain in Nepal. However, understanding the nuances of these journeys requires moving beyond a simple tally of who migrated and who stayed. Examining the factors that shaped their choices, including the challenges and opportunities encountered, provides crucial insights into the broader phenomenon of healthcare worker migration. The “brain drain” phenomenon poses a significant challenge to Nepal's healthcare system, as the country struggles to retain its skilled workforce, which is essential to meet the growing healthcare needs of its population (Pokharel et al., 2024). A complex interplay of push and pull factors drives the migration of healthcare professionals from Nepal to more developed nations. Push factors include inadequate healthcare infrastructure, low wages, challenging working conditions, and limited career advancement opportunities within Nepal (International Labour Organization, 2017; Toyin-Thomas et al., 2023). Conversely, pull factors encompass higher salaries, improved working conditions, and enhanced professional development prospects in destination countries (Munikar & Thapa, 2019; Thapa & Shrestha, 2017). This exodus of skilled healthcare workers exacerbates the already critical shortage; as of 2021, Nepal's nursing workforce stood at just 3.5 per 1,000, highlighting the urgent need for a robust healthcare workforce (WHO, 2021). The situation is further compounded by the phasing out of mid-level health worker training programs under the Council for Technical Education and Vocational Training (CTEVT), Nepal's governing body responsible for technical and vocational education (Basnet & Karki Pyakurel, 2023). This vocational training was tailored to produce a mid-level technical nursing workforce with the necessary knowledge and skills to fulfill the country's demand for nursing. Furthermore, the recruitment agreement between the UK and Nepalese governments to employ Nepali nurses amid Nepal's classification as one of the “red list” countries, featuring a severe workforce shortage, presents ethical concerns (Department of Health and Social Care, 2022; Ghimire, Qiu, Neupane, et al., 2024; National Health Service, 2023). The WHO prohibits active recruitment of health workers from red-listed countries facing an acute workforce shortage unless a government-to-government agreement exists (National Health Service, 2023; WHO, 2023). In this context, the complexities surrounding the migration of Nepali nurses, particularly in light of these ethical considerations, necessitate a deeper understanding of their motivations and experiences beyond the initial decision to migrate.

Adding to these complexities are the bureaucratic hurdles faced by internationally educated nurses, including those from Nepal. A significant hurdle in the migration journey for internationally educated nurses is navigating the complex visa requirements of destination countries. High per-capita income countries such as Australia, Canada, the United Kingdom, and the United States attract large numbers of registered nurses (RNs) from developing countries (Kurup et al., 2023; Walani, 2015). While the specific procedures vary, common prerequisites include credential evaluation, language proficiency tests, and licensure exams. For instance, nurses seeking to work in the United States typically need to obtain a visa screen certificate from the Commission on Graduates of Foreign Nursing Schools (CGFNS) and pass the National Council Licensure Examination (NCLEX) for Registered Nurses (RN) exam (CGFNS, 2025). In Canada, nurses must undergo a skills assessment by a designated regulatory body and demonstrate proficiency in English or French, often through the Express Entry system or Provincial Nominee Programs (Canadian Immigration Law Firm, 2023). Similarly, Australia requires a skills assessment from the Australian Nursing and Midwifery Accreditation Council (ANMAC) and an Expression of Interest through the Skilled Migration system (Nursing and Midwifery Board of Australia, 2024). The United Kingdom, facing a nursing shortage, often recruits internationally educated nurses through its Skilled Worker visa, requiring registration with the Nursing and Midwifery Council (National Health Service Employers, 2024). These bureaucratic processes, while intended to ensure the quality and safety of healthcare, can present significant challenges and delays for aspiring migrant nurses.

Despite these challenges, previous research on Nepali nurse migration has predominantly focused on premigration factors, such as economic incentives, career aspirations, and perceived deficiencies in the domestic healthcare system (Munikar & Thapa, 2019; Poudel et al., 2018). While these studies provide valuable insights, they often overlook the dynamic nature of migration decisions and the complexities of the postmigration experience. To fully understand this phenomenon, adopting a longitudinal perspective that tracks nurses’ journeys over time is crucial. This study addresses these gaps in the literature by employing a longitudinal qualitative approach to explore the evolving motivations, expectations, and experiences of a cohort of Nepali nursing graduates over 1 year following their graduation. Specifically, this research seeks to answer the following questions:

- How do the lived experiences of migrant Nepali nurses align with or differ from their premigration expectations?
- What strategies do migrant nurses employ to overcome challenges, foster integration, and build successful careers in their new countries?
- What factors influence the decision of some Nepali nursing graduates to remain in Nepal?

By addressing these questions, this study aims to provide a more nuanced understanding of the complex landscape of nurses’ migration from Nepal.

## Method

### Research Design

This study employed a descriptive qualitative methodology, following the principles outlined by Sandelowski (2000). Semi-structured interviews, guided by open-ended questions, provided a platform for participants to share their personal and professional journeys in depth. The questions were formulated based on a comprehensive literature review on healthcare worker migration and the research team members’ lived experiences as migrant nurses.

### Participants

This longitudinal study followed the same cohort of 17 Nepali nursing graduates who participated in the initial study one year prior (Ghimire, Qiu, Thapa, et al., 2024). Participants were recruited during their final year of study from two prominent tertiary institutions in Bharatpur, a major urban center in Nepal's Chitwan district. This institution was chosen due to its reputation as a leading nursing education provider in the region. The initial study, as detailed in Ghimire, Qiu, Thapa, et al. (2024), employed purposive sampling to recruit participants who were in their final year of a Bachelor of Science in Nursing (BSc Nursing) or a Bachelor of Nursing Science program.

For this follow-up study, the 17 participants were contacted via phone and email using the contact information provided during the initial study, and all agreed to participate. All 17 participants had expressed an intention to migrate during the initial study. However, their preferred destinations and specific migration plans varied. At the time of the initial interviews, their preferred destination countries included the United States, the United Kingdom, Australia, and Germany. It is important to note that participants were not recruited based on their intended destination countries. Of these, 12 had successfully migrated within 1 year of graduation, primarily to destinations such as the United States, United Kingdom, and Australia. The remaining five participants continued to reside in Nepal at the time of the follow-up interviews. While these five participants initially intended to migrate, their plans had either been delayed or changed due to various factors, including bureaucratic hurdles, evolving personal priorities, or unexpected opportunities within Nepal, as explored in the Results section. Table 1 provides a summary of the participants’ demographic information and migration status.

**Table 1.** Summary of Demographic Information.

Participant	Age	Gender	Premigration destination preference (2023)	Current location (2024)	Migration status (2024)
P1	24	Female	Australia	Australia	Migrated
P2	23	Female	The United States	The United States	Migrated
P3	22	Female	The United States	The United States	Migrated
P4	23	Female	Australia	Nepal	Remained
P5	25	Female	Australia	Nepal	Remained
P6	22	Female	Germany	Nepal	Remained
P7	22	Female	Australia	Nepal	Remained
P8	22	Female	The United States	The United States	Migrated
P9	21	Female	The United States	The United States	Migrated
P10	23	Female	Australia	Australia	Migrated
P11	22	Female	Australia	Nepal	Remained
P12	23	Female	Australia	Australia	Migrated
P13	26	Female	The United States	The United States	Migrated
P14	24	Female	The United States	The United States	Migrated
P15	23	Female	The United Kingdom	The United Kingdom	Migrated
P16	23	Female	Australia	Australia	Migrated
P17	24	Female	Australia	Australia	Migrated

### Data Collection

Data collection involved two rounds of semi-structured interviews conducted one year apart by the first author (A.G.). The initial interviews, held in June 2023, just before graduation, explored participants’ premigration intentions, perceived push and pull factors, expectations of work and life abroad or reasons for staying in Nepal, and their hopes for the future. A 1-year follow-up period was chosen to allow sufficient time for participants to enact their migration intentions while still being recent enough to recall the factors influencing their decisions. The follow-up interviews, conducted in July 2024, delved deeper into participants’ experiences since the initial interview. For those who had migrated, questions focused on the realities of their work environments, cultural adjustment challenges, support systems, and any shifts in their perceptions of migration. For those who remained in Nepal, questions centered on their experiences in the domestic healthcare system, reasons for not migrating (or delaying migration), and evolving career aspirations (see Table 2). The interviews, lasting approximately 60–90 min each, were conducted in person for participants in Nepal and via video call for those who had migrated.

**Table 2.** Sample Interview Questions.

Question number	Stage 1: Interview questions (2023)	Stage 2: Interview questions (2024)—migrated nurses	Stage 2: Interview questions (2024)—nonmigrant nurses
1	Based on your observations or insights into healthcare systems abroad, what key modifications would you introduce to Nepal's healthcare framework?	Reflecting on your initial expectations of migrating, how has the reality of living and working abroad compared? What specific aspects have been surprising or different?	What were your initial motivations for considering migration, and how have they evolved over the past year? What factors influenced your decision to remain in Nepal or postpone your plans?
2	Could you recount a specific occasion where the structure of Nepal's healthcare system adversely affected your learning or practical training as a nursing student?	Describe the most significant challenges and rewards you've experienced since migrating. How have these shaped your overall perspective on your decision?	What challenges and rewards have you encountered in pursuing your nursing career in Nepal? How have these experiences influenced your career goals and aspirations?
3	Describe your ideal nursing career. Do you consider such a career achievable in Nepal, or do you see it more likely through overseas opportunities?	How have your personal priorities and career goals shifted since your initial decision to migrate? Have there been any unexpected opportunities or challenges that influenced this evolution?	What unexpected opportunities or challenges have you encountered in your nursing career in Nepal? Have these experiences led you to reconsider your initial decision to stay?
4	How have societal attitudes or stereotypes about nursing in Nepal shaped your career goals, particularly regarding working within the country versus abroad?	In what ways has your work environment in [destination country] met or not met your expectations? What specific aspects have been fulfilling or disappointing?	How does your current work environment in Nepal compare to what you envisioned during your studies? What aspects have been surprising or different?
5	Reflect on your personal ambitions and how they align or diverge from the conventional career trajectory of a nurse in Nepal.	Have you experienced any difficulties related to cultural adjustment, social integration, or building a sense of belonging in your new country? How have you navigated these challenges?	Have you experienced any challenges in finding a sense of belonging or purpose within your current work environment or community in Nepal? If so, how have you addressed these challenges?
6	How have recent socio-political developments in Nepal influenced your views on the stability and prospects of the nursing profession in the country?	What strategies or resources have been most helpful in navigating your transition and fostering a sense of belonging in your new country?	What strategies or resources have you utilized to build your career and find professional satisfaction in Nepal? What additional support would be beneficial for nurses choosing to stay and work in the country?
7	Compare the economic aspects of nursing in Nepal with those of opportunities abroad. How do these considerations affect your career planning?	How does the reality of your financial situation abroad compare to your initial expectations? Have there been any unexpected financial challenges or benefits?	How do you perceive the financial aspects of nursing in Nepal now, compared to your views a year ago? Has your financial situation influenced your decision to stay or reconsider migration?
8	What are your thoughts on the wage disparity between nurses and doctors in Nepal, and how does this gap affect your view of the nursing profession?	How has the experience of working abroad impacted your professional identity as a nurse? Do you feel your skills and contributions are valued in your current role?	Has your perception of the nursing profession in Nepal changed over the past year? Do you feel your skills and contributions are valued in your current role?
9	Share an instance where the healthcare system's hierarchy influenced your decisions or role as a nursing student or professional.	Reflecting on your initial reasons for choosing to migrate, have those reasons changed or evolved? If so, how?	How have your relationships with family, friends, or mentors influenced your decision to stay in Nepal and pursue your career there?
10	Have you encountered or observed situations that compromised your safety or that of your peers as nursing students or practitioners? How did such experiences shape your perspective on pursuing a nursing career in Nepal?	How have you maintained connections with your family and friends back in Nepal? What role have these relationships played in your overall well-being and adjustment abroad?	What are your future career aspirations, and do you see yourself continuing to build your career in Nepal or reconsidering migration in the future?
11	How does your family and social circle's perception of nursing impact your decision to work in Nepal or explore opportunities abroad?	What advice would you give to current nursing students in Nepal who are considering migration? What factors should they weigh in their decision-making process?	What changes or improvements would you like to see in the Nepali healthcare system to better support nurses and encourage them to stay and contribute to the country's healthcare workforce?

### Data Analysis and Rigor

The first author transcribed all audio-recorded interviews verbatim and translated them into English where necessary. A second author independently reviewed a random selection of interview transcripts to ensure accuracy. The study followed Braun and Clarke’s (2023) guidelines for reflexive thematic analysis. The research team engaged in an iterative process of data familiarization, code generation, and theme development. The sample size was determined by the number of participants in the initial study (Ghimire, Qiu, Thapa, et al., 2024), as the aim was to follow the same cohort longitudinally.

Initial open coding involved identifying salient excerpts and assigning descriptive codes that captured the essence of the participants’ experiences and perspectives. These initial codes were then systematically organized into broader categories based on shared conceptual meanings, through discussions among the research team. These categories were refined and condensed into overarching themes and subthemes through ongoing discussion and consensus-building among the research team, ensuring that the final themes accurately reflected the underlying patterns and relationships within the data. This process involved constant comparison and contrast of codes, categories, and themes to ensure consistency and coherence.

Two researchers independently coded a subset of the data to enhance rigor and trustworthiness, resolving discrepancies through collaborative discussion. An audit trail documented the evolution of codes, categories, and themes, ensuring transparency and replicability. Member checking was conducted by sharing preliminary findings with participants to validate interpretations and ensure the accurate representation of their experiences. Additionally, detailed descriptions of participants’ demographic and professional characteristics are included in this article to enhance the transferability of the findings.

Table 3 provides a detailed example of the data analysis process, showcasing the progression from data extracts to initial codes, refined codes, subthemes, and finally, the overarching themes. This approach aligns with Rahimi and Khatooni (2024) who emphasize code and thematic saturation over data saturation, which was deemed more appropriate given the participants’ diverse and evolving experiences. While no new codes or themes emerged during the analysis of the final interviews, this approach prioritizes the depth and richness of the analysis over the sheer quantity of data collected, acknowledging that individual stories might still vary with additional participants.

**Table 3.** Illustrative Examples of the Coding Process.

Data extract	Initial codes	Refined codes	Subthemes	Themes
“Honestly, by the time graduation party rolled around, it felt like half our class had their plane tickets already booked. It was like everyone had caught the migration fever.” (P16)	Migration fever, graduation, plane tickets, everyone going	Normalized migration, collective mindset, social pressure, common aspiration	Migration as a cultural norm and a path to success	Balancing dreams, pragmatism, and the emotional costs of migration
“Sometimes I miss the chaos of Kathmandu, just a little. But then I get my paycheck and remember … there's a reason so many of us left.” (P8)	Missing home, paycheck, reason for leaving	Emotional cost, financial benefit, pragmatism, trade-off	The bittersweet reality: balancing aspirations with emotional costs	Balancing dreams, pragmatism, and the emotional costs of migration
“I had everything ready—transcripts, language certificates, even a job offer lined up. But then the visa process turned into a nightmare. […] I just got so fed up, I decided it wasn't worth the headache.” (P1)	Visa process, nightmare, delays, fees, giving up	Bureaucratic hurdles, frustration, disillusionment, barriers to migration	Bureaucratic barriers as a deterrent	Evolving priorities, unexpected opportunities, and challenging the migration narrative
“At first, I felt like a failure for not going abroad like everyone else. But then I got this position at a specialized clinic […] I'm making a real difference.” (P7)	Failure, specialized clinic, making a difference	Initial self-doubt, unexpected opportunity, professional fulfillment	Unexpected fulfillment within Nepal	Evolving priorities, unexpected opportunities, and challenging the migration narrative
“I came expecting the ‘big bucks’ everyone talks about … Feels like all this struggle is for a tiny bit of extra money.” (P13)	Big bucks, struggle, little extra money	Financial expectations, disillusionment, cost of living, minimal gains	The financial gap: remuneration disparity	Unmet expectations, aspirations, and the struggles of migration
“Back home, I dreamed of working in a state-of-the-art ICU … it feels like I'm going backward.” (P2)	Dream ICU, outdated ward, going backward	Unmet aspirations, professional stagnation, lack of growth, skill underutilization	Professional stagnation: unfulfilled aspirations	Unmet expectations, aspirations, and the struggles of migration
“At first, the language barrier felt overwhelming. But I enrolled in an intensive course […] now I understand their accent and the slang patients use. It opened so many doors.” (P9)	Language barrier, intensive course, opened doors	Overcoming challenges, language acquisition, professional integration	Language as a bridge to success	Strategies for integration, resilience, and belonging
“I was lucky to find a senior nurse at my hospital who also came from Nepal. She helped me navigate the system, unwritten rules of the ward […] having that guidance made all the difference.” (P12)	Senior nurse, guidance, made a difference, from Nepal	Mentorship, support, navigating new system, shared background	The guiding hand of mentorship	Strategies for integration, resilience, and belonging
“We have a lot more autonomy back home whereas here there is a clear boundary regarding our scope of practice.” (P4)	Autonomy, scope of practice, boundary	Professional adjustment, different roles, adaptation, role limitations	Adjusting to different healthcare systems	Navigating transition: the experience of clinical practice abroad
“The exposure I have had working back home, where we are completely thrown into the deep end without much support, in hindsight, it has worked in my favor. My colleagues and managers here value my experience.” (P10)	Exposure, experience valued, thrown in the deep end, valued by colleagues and managers	Prior experience, unexpected advantage, skill recognition, resilience	Leveraging prior experiences and skills	Navigating transition: the experience of clinical practice abroad

### Ethical Considerations

Ethical approval was obtained from the Nepal Health Research Council (NHRC-133/2023). Prior to participation, all potential participants received a comprehensive information sheet detailing the study's purpose, procedures, potential risks and benefits, and their right to withdraw at any time without consequence. Written informed consent was obtained from all participants who agreed to be part of the study.

## Results

This study identified five overarching themes from the data, encompassing 13 subthemes. Table 4 provides an overview of these findings.

**Table 4.** Themes and Subthemes.

Theme	Subtheme
Balancing dreams, pragmatism, and the emotional costs of migration	Migration as a cultural norm and a path to success The bittersweet reality: balancing aspirations with emotional costs
Evolving priorities, unexpected opportunities, and challenging the migration narrative	Bureaucratic barriers as a deterrent Shifting priorities and the power of personal relationships Unexpected fulfillment within Nepal
Unmet expectations, aspirations, and the struggles of migration	The financial gap: remuneration disparity Professional stagnation: unfulfilled aspirations The cost of belonging: cultural adjustment struggles
Strategies for integration, resilience, and belonging	Language as a bridge to success The guiding hand of mentorship Maintaining ties, building community
Navigating transition: the experience of clinical practice abroad	Adjusting to different healthcare systems Leveraging prior experiences and skills

### Theme 1: Balancing Dreams, Pragmatism, and the Emotional Costs of Migration

For Nepali nursing graduates, the decision to migrate is far from a simple equation. It is a dynamic process that balances dreams of professional advancement with pragmatic considerations and the emotional toll of leaving one's homeland.

#### Subtheme 1.1: Migration as a Cultural Norm and a Path to Success

The narrative of migration as a path to success is deeply ingrained in Nepal's social fabric.

Honestly, by the time graduation party rolled around, it felt like half our class had their plane tickets already booked. It was like everyone had caught the migration fever. (P16)

This cultural expectation can influence individual decision-making, as evidenced by another participant's statement:

It wasn’t even a question of “if” I would leave, but “where” […] This was a dream harbored from the moment I stepped into my nursing school back home. (P10)

#### Subtheme 1.2: The Bittersweet Reality: Balancing Aspirations With Emotional Costs

While the allure of working abroad is undeniable, the reality of migration often brings a mix of emotions, including fulfillment, regret, and longing. One participant reflected on the bittersweet nature of their experience:

Most of my fellow graduates have gone overseas. We always talked about what our lives would be like and how working in modern, first-world hospitals would feel like. This has become a reality for many of us; it is a bittersweet feeling. (P2)

This emotional complexity is further highlighted in another participant's statement:

Sometimes I miss the chaos of Kathmandu, just a little. But then I get my paycheck and remember … there’s a reason so many of us left. (P8)

### Theme 2: Evolving Priorities, Unexpected Opportunities, and Challenging the Migration Narrative

This theme encapsulates the experiences of graduates who initially intended to migrate but ultimately remained in Nepal. Their stories defy simplistic notions of “brain drain,” highlighting individual agency, evolving priorities, and discovering unexpected career fulfillment within their home country.

#### Subtheme 2.1: Bureaucratic Barriers as a Deterrent

For some graduates, the dream of migrating was thwarted not by a lack of desire but by the complexities and frustrations of immigration systems.

I had everything ready—transcripts, language certificates, even a job offer lined up. But then the visa process turned into a nightmare. Forms, delays, fees […]. I spent months in limbo. Eventually, I just got so fed up, I decided it wasn’t worth the headache. (P1)

The visa process was like a maze with no exit. Every time I thought I was close, another hurdle would appear. It drained my energy, my savings, and my hope. (P7)

#### Subtheme 2.2: Shifting Priorities and the Power of Personal Relationships

Life's unexpected turns can also alter migration trajectories.

Honestly, meeting my husband changed everything. Before, I thought nothing would keep me in Nepal. But he built a business here, his family is close […] suddenly leaving didn’t make sense anymore. There are good nursing jobs here too, once you look outside the big cities. (P4)

My family means the world to me. The thought of being an ocean away from them, especially my aging parents, became unbearable. I realized that no amount of money or career success could replace their presence in my life. (P6)

#### Subtheme 2.3: Unexpected Fulfillment Within Nepal

Contrary to Nepal's pervasive narrative of limited opportunities, some nurses found unexpected professional fulfillment within their home country.

At first, I felt like a failure for not going abroad like everyone else. But then I got a scholarship to do my Masters degree and a permanent full-time job at a government hospital. The work is challenging, the pay is decent, and most importantly, my education costs are covered. I also feel like I’m making a real difference—being where I am most needed. (P7)

The turning point for me was realizing that I could make a real impact right here, in the very village where I was born. I found a job at the local hospital. Sure, challenges are there, but aren’t they everywhere? The gratitude from the patients … it’s something you can’t put a price on. I don’t know what the future holds, but for now, I’m content. (P5)

### Theme 3: Unmet Expectations, Aspirations, and the Struggles of Migration

While the prospect of a brighter future abroad motivates many Nepalese nurses, the realities of migration often bring unforeseen challenges and emotional costs. Once characterized by hope and anticipation, the migration journey can become marked by disillusionment as the perceived opportunities and experiences fail to match the initial aspirations.

#### Subtheme 3.1: The Financial Gap: Remuneration Disparity

One of the most common discrepancies between expectations and reality was observed in the realm of financial gain.

I came expecting the “big bucks” everyone talks about. Sure, the pay is better than back home, but with the cost of living here? I’m barely saving more than I used to. Feels like all this struggle is for a tiny bit of extra money. (P1)

The money was good, at least on paper. But then you factor in rent, taxes, the cost of just […] existing here. Suddenly, that dream salary does not seem so big anymore. (P13)

#### Subtheme 3.2: Professional Stagnation: Unfulfilled Aspirations

Another common point of disillusionment was the lack of professional growth opportunities.

Back home, I am an ICU-trained nurse and dreamed of working in a state-of-the-art ICU. Instead, I’m stuck being a ward nurse, unable to utilize my skills. I thought I’d move forward here, but my career is going backward. (P16)

I feel like my skills are being wasted here […] I’m capable of so much more, but I’m stuck in a role that doesn’t challenge me or allow me to grow. (P11)

#### Subtheme 3.3: The Cost of Belonging: Cultural Adjustment Struggles

Beyond the professional realm, the emotional toll of cultural adjustment emerged as a significant challenge.

Nobody prepared me for the loneliness. People are friendly on the surface, but I still haven’t found a real friend. And at work, it’s these subtle ways of being that I don’t quite get right, it makes you feel constantly like an outsider. (P3)

The hardest part is the silence. Back home, there’s always noise, laughter, family around. Here, I come home to an empty apartment. It’s a different kind of quiet … a lonely one. (P8)

### Theme 4: Strategies for Integration, Resilience, and Belonging

While migration presents numerous challenges, Nepalese nurses who successfully navigated their transition into new healthcare systems demonstrated remarkable adaptability and resilience.

#### Subtheme 4.1: Language as a Bridge to Success

Language proficiency emerged as a crucial factor in fostering professional integration and enhancing patient care.

At first, the language barrier felt overwhelming. But I enrolled in an intensive course, practiced with colleagues […] now I understand their accent and the slang patients use. It opened so many doors. (P9)

Language isn’t just about words; it’s about understanding the culture. Learning the local expressions, the jokes … it helps you connect with people, build trust. It’s made me a better nurse, not just a more fluent one. (P14)

#### Subtheme 4.2: The Guiding Hand of Mentorship

Navigating a new workplace culture and unfamiliar systems can be daunting. Finding mentors who have successfully navigated similar transitions can be invaluable.

I was lucky to find a senior nurse at my hospital who also came from Nepal. She helped me navigate the system, unwritten rules of the ward […] having that guidance made all the difference. (P12)

Having a mentor was invaluable. They’ve been there, they know the pitfalls, they can guide you towards the opportunities. I wouldn’t have survived my first year without mine. (P10)

#### Subtheme 4.3: Maintaining Ties, Building Community

Preserving connections to their home country while building new relationships within the host country emerged as a crucial strategy for fostering belonging.

Video calls with my family back home are a lifeline. But also, I joined a Nepali cultural group here—having that shared connection, celebrating festivals, it reminds me I’m not alone. (P15)

The online Nepali community has been a lifesaver. We share tips, vent about our struggles, even celebrate festivals together virtually. It is like having a piece of home right here with me. (P9)

### Theme 5: Navigating Transition: The Experience of Clinical Practice Abroad

This theme explores the experiences of Nepalese nurses as they transition into clinical practice within foreign healthcare systems.

#### Subtheme 5.1: Adjusting to Different Healthcare Systems

The shift to a new healthcare system often involved learning unfamiliar protocols, technologies, and professional roles.

We have a lot more autonomy back home whereas here there is a clear boundary regarding our scope of practice. (P1)

While the reduced autonomy might initially feel restrictive, it provides clearer expectations and potentially a safer working environment for new nurses.

I wasn’t aware of my rights, the leave I was entitled to, and the breaks during the shifts. Here it is regulated, and we are encouraged to voice our opinion. Back home, we just worked tirelessly. (P3)

#### Subtheme 5.2: Leveraging Prior Experiences and Skills

Many nurses found that their prior experience in Nepal had equipped them with valuable skills and resilience that aided their integration into new workplaces.

The rigorous 4 years we spent in our undergraduate degree is so worth it. Initially, I felt I would lack in many aspects, but I feel I am no less than those nurses educated here. (P13)

The exposure I have had working back home, where we are completely thrown into the deep end without much support, in hindsight, it has worked in my favor. My colleagues and managers here value my experience. (P14)

## Discussion

This study explored the multifaceted experiences of Nepalese nurses as they navigated the complexities of migration and professional integration abroad. These experiences reveal the tension between pursuing professional advancement and the emotional and cultural challenges of leaving home. Key insights emerged, highlighting the motivations behind migration, the unexpected realities encountered, and the critical role of language acquisition, mentorship, and previous training in shaping their integration into new healthcare systems. However, a deeper understanding of these motivations necessitates revisiting the initial aspirations expressed by these nurses prior to migration. In our first study (Ghimire, Qiu, Thapa, et al., 2024), all 17 participants, including those who eventually remained in Nepal, unanimously expressed an intention to migrate, driven by factors such as limited opportunities for professional growth, inadequate compensation, and socio-political instability within Nepal. These initial intentions, while seemingly homogenous, masked a more nuanced reality that unfolded over the course of a year.

This longitudinal study provides a nuanced counterpoint to those prevailing narratives on nurse migration, which often portray it as a unidirectional flow driven primarily by economic incentives and a desire for improved working conditions (Konlan et al., 2023; Rolle Sands et al., 2020). While our initial study corroborated these factors as significant motivators (Ghimire, Qiu, Thapa, et al., 2024), revealing a unanimous intention to migrate among 17 Nepali nursing students, the follow-up interviews, one year later, painted a far more complex picture. The lived experiences of these nurses, both those who migrated and those who remained in Nepal, underscore the dynamic and often unpredictable nature of migration journeys. Challenging the simplistic “brain drain” discourse, our findings reveal that migration decisions are not static outcomes but rather evolve in response to a confluence of shifting personal priorities, unexpected opportunities, and bureaucratic hurdles. For instance, the subtheme “Bureaucratic Barriers as a Deterrent” vividly illustrates how immigration policies and visa complexities can significantly alter migration trajectories, a dimension often overlooked in studies focusing solely on premigration intentions. This study identified the visa process as a major obstacle for many nurses. While 12 out of 17 nurses successfully migrated, the difficulties encountered during the visa application process, such as complex paperwork, long processing times, and high fees, highlight the significant impact of immigration policies on individual aspirations. It is crucial to recognize that the challenges faced in Nepal are emblematic of a larger global framework of immigration regulations that govern the mobility of healthcare professionals. This framework particularly affects the flow of skilled healthcare professionals from low- and middle-income countries to high-income countries (Ramani & Rutkofsky, 2021; Smith et al., 2024). While aiming to manage migration flows and protect the interests of receiving countries, this system inadvertently creates barriers for qualified nurses seeking to work abroad, impacting both their career trajectories and the global healthcare workforce (Adhikari & Grigulis, 2013; Masselink & Jones, 2014). Furthermore, the narratives of those who remained in Nepal provide a crucial counter-narrative to the assumption that migration is the only pathway to professional fulfillment. These nurses discovered unexpected opportunities for growth and meaning within their home country, highlighting the importance of considering the agency and resilience of individuals within broader structural forces. This aligns with the scholarship on transnationalism, which emphasizes the multidirectional and circulatory nature of migration and the potential for individuals to maintain meaningful connections and find fulfillment in both sending and receiving countries (Kasun et al., 2022; Lacroix et al., 2024). This study elucidates the complex dynamics of nurses’ motivations and experiences throughout their migration journeys, enhancing the discourse on the migration phenomenon. It emphasizes the intricate relationship between personal aspirations, systemic limitations, and the unpredictable nuances that shape individual life trajectories.

The allure of a better life abroad, fueled by dreams of professional advancement and financial gain, serves as a potent motivator for many Nepalese nurses. However, the decision to migrate is far from straightforward, often entailing a complex negotiation between personal aspirations and the emotional weight of leaving behind family, community, and cultural familiarity. The “bittersweet” sentiment expressed by participants encapsulates this tension, highlighting the sacrifices inherent in pursuing dreams of a brighter future. The allure of “modern, first-world hospitals” and the pervasive “migration fever” underscores how deeply ingrained the narrative of migration as a pathway to success is within Nepali society. This phenomenon aligns with the “cumulative causation” theory, where the success stories of early migrants fuel a self-perpetuating cycle of outward mobility (International Organization for Migration, 2024; Massey, 1990). Individual aspirations and familial expectations further complicate the decision to migrate, particularly within Nepal's collectivist culture (Wali & Renzaho, 2018). Pursuing personal ambitions can clash with deeply held societal norms and family obligations, creating a sense of guilt and internal conflict. While migration can be seen as an expression of agency, particularly for women navigating limited opportunities and patriarchal structures in Nepal (Dahal et al., 2022), it is crucial to acknowledge the potential negative consequences of “brain drain” on the source country's healthcare system.

On the other hand, the narratives of participants who chose to remain in Nepal offer a compelling counterpoint to the dominant narrative that often frames migration as the only avenue for professional advancement in the healthcare field. These nurses’ experiences highlight the potential for “brain circulation” or “brain gain” within Nepal, where skilled professionals find fulfilling and impactful career opportunities within their home country. This perspective shifts the focus from a unidirectional loss of talent to a more dynamic understanding of how skills and knowledge can be developed and utilized within national borders. This finding suggests that addressing the underlying “push” factors, such as limited career advancement opportunities, inadequate remuneration, and perhaps most importantly, a lack of recognition for nurses’ expertise and contributions, could be key to retaining skilled nurses in Nepal and fostering a more robust domestic healthcare system (Hashish & Ashour, 2020; Konlan et al., 2023). While “reverse brain drain” programs, which encourage health professionals to return from high-income countries through investment in research and technology, have shown promise in countries like Thailand, Taiwan, and South Korea (Dia, 2022; Sahay, 2014), their replicability in other contexts remains uncertain (Kamarulzaman et al., 2022). Nonetheless, for those migrant aspirants, the frustration and disillusionment due to the complexities of visa processes highlight the significant impact immigration policies can have on individual aspirations and the global distribution of healthcare workers. This resonates with the findings of WHO (2024) that emphasized the role of immigration policies in shaping healthcare worker mobility. The “airlift” of migrant nurses during COVID-19 in Australia, bypassing quarantine requirements, highlights the self-interested motives of developed countries that readily adapt migration policies to their immediate needs (Buchan et al., 2022).

Notably, migrant nurses discovered that their experiences differed from their expectations of premigration. While higher salaries abroad may seem attractive, the realities of living expenses, taxes, and unforeseen costs can significantly diminish the perceived financial gains aligning with research on the “myth of the economic migrant,” which questions the assumption that individuals migrate solely for economic reasons (de Haas, 2021; Goodfellow, 2023). Our findings suggest that financial motivations are often intertwined with other factors, such as professional aspirations and social mobility, and that the actual economic benefits of migration may not always meet expectations. Furthermore, the frustration expressed by participants over limited professional growth opportunities highlights a critical challenge faced by many migrant nurses. This phenomenon is well documented in the literature on migrant nurses, with studies reporting underutilization of skills, limited access to professional development, and difficulties in obtaining recognition for foreign qualifications (Kamau et al., 2022; Kurup et al., 2024; Smith et al., 2022). This underutilization of skills can be seen as a form of “status inconsistency,” where individuals occupy a lower social position than their education and qualifications would suggest (Milner et al., 2017). The experience of diminished professional standing and the emotional strain of adapting to a new culture can result in a lasting feeling of being an “outsider.” This phenomenon can be conceptualized as acculturative stress (Adebayo et al., 2021; Zlotnick et al., 2024). Additionally, experiences of “otherness,” like microaggressions and implicit biases in the workplace, contribute to a sense of marginalization and exclusion.

Despite these systemic challenges, the narratives of Nepalese migrant nurses highlight a dynamic interplay between personal agency and structural support in navigating the complexities of integration. The transformative power of language learning, described as “opening so many doors,” underscores its role beyond mere functional communication. Mastering the nuances of medical terminology, accents, and informal language allows migrant nurses to connect with patients and colleagues, fostering trust and understanding (Gerchow et al., 2021). The importance of mentorship and community-building further underscores the multifaceted nature of integration. The guidance provided by experienced colleagues, particularly those from a shared cultural background, helps nurses navigate the often unspoken norms and expectations of their new workplaces. However, reliance on “luck” in finding a mentor highlights a potential gap in institutional support. Establishing formal mentorship programs for migrant nurses could accelerate their integration and harness their valuable cultural and linguistic knowledge, fostering a more inclusive and culturally competent healthcare environment (Červený et al., 2022).

The narratives of Nepalese nurses in this study defy the prevailing notion of challenging adaptation by exploiting the global interconnectedness in nursing education. They reported a smoother transition than anticipated, leveraging their prior experiences and training in Nepal to navigate new professional landscapes. The nurses’ ability to navigate this transition can be attributed to a combination of individual agency and the unique characteristics of their training in Nepal. The rigorous nursing education, often characterized by hands-on experience in a limited resource setting, appears to have fostered a sense of resourcefulness and adaptability that proved invaluable in their new workplaces (Peel et al., 2021). While some nurses initially found the reduced autonomy and stricter hierarchies in their new workplaces challenging, they also recognized the benefits of clearer expectations, regulated work hours, and rights, contrasting with Nepal's often demanding and less structured work environments (Thapa et al., 2022). This suggests that different healthcare systems’ perceived advantages and disadvantages are subjective and can vary depending on individual preferences and experiences. The positive reception of their prior experiences in Nepal further challenges the deficit-based view often applied to migrant nurses from developing countries (Korzeniewska & Erdal, 2019; Newton et al., 2012).

## Strengths and Limitations

The longitudinal design of this study, with interviews conducted both pre- and postgraduation, offers a unique perspective on the evolving motivations and experiences of Nepali nursing graduates. This approach allowed for a deeper understanding of the dynamic nature of migration decision-making and the complexities of adaptation. The inclusion of both migrant and nonmigrant nurses further enriched the data, providing a more comprehensive picture of the diverse pathways pursued by graduates. However, the study also has limitations. The relatively small sample size may limit the generalizability of the findings. The focus on two private nursing colleges in Nepal may not fully represent the experiences of nurses from other regions or educational institutions. Additionally, the study did not explore the experiences of nurses who migrated to destinations beyond the three countries mentioned, potentially limiting the understanding of the diverse migration pathways and challenges faced by Nepali nurses. Future research could address these limitations by expanding the sample size and geographic scope and exploring the long-term trajectories of migrant nurses.

## Conclusion

This study provides a nuanced perspective on the migration journeys of Nepali nursing graduates, moving beyond a simplistic “brain drain” analysis to reveal the dynamic interplay of individual aspirations, structural constraints, and unexpected opportunities that shape their trajectories. While the allure of professional advancement and financial gain abroad remains a powerful motivator, the narratives of these nurses, both those who migrated and those who stayed, highlight the significant influence of evolving personal priorities, the often-unforeseen challenges of navigating complex immigration systems, and the potential for discovering fulfilling career paths within Nepal. These findings underscore the need for policies that address the root causes of nurse migration, not just by focusing on economic incentives but also by fostering an environment within Nepal that supports the professional growth and well-being of its nursing workforce. Furthermore, the study emphasizes the importance of ethical recruitment practices and comprehensive support systems for migrant nurses in destination countries. Ultimately, a more holistic understanding of nurse migration, one that acknowledges both the structural forces at play and the agency of individuals in making choices that align with their evolving aspirations, is crucial for developing strategies that benefit both source and destination countries.
